# Editorial: Strategies for workplace design to reduce obesity and metabolic risks

**DOI:** 10.3389/fpubh.2026.1866218

**Published:** 2026-06-25

**Authors:** Mohammad Hossein Ebrahimi, Mojtaba Keikha, Mostafa Dianati

**Affiliations:** 1 Environmental and Occupational Health Research Center, Shahroud University of Medical Sciences, Shahroud, Iran; 2 Department of Public Health, Sirjan School of Medical Sciences, Sirjan, Iran; 3 Department of Complex Genetics and Epidemiology, Maastricht University Medical Centre, Maastricht, Netherlands

**Keywords:** cardiometabolic risk, metabolic syndrome, occupational health, shift work, workplace design

The modern occupational landscape is increasingly characterized by sedentary behaviors, circadian disruption, and constrained access to health-promoting resources, collectively contributing to the rising prevalence of obesity and metabolic syndrome among working adults. Traditional wellness programs often fall short because they address individual behavior in isolation from the structural and environmental determinants of health. Recent occupational health research underscores the necessity of redesigning workplace environments to actively support metabolic health through integrated nutritional infrastructure, circadian-aligned scheduling, precision health surveillance, and musculoskeletal-metabolic promotion. By synthesizing findings from five recent occupational cohort studies, this review outlines evidence-based strategies for workplace design that can systematically reduce obesity and cardio metabolic risk across diverse professional settings.

The physical and logistical design of workplace food environments plays a decisive role in shaping employee dietary patterns and energy balance. Work-related meal delays and frequent skipping were strongly inversely associated with healthy eating behaviors among healthcare providers, with reliance on takeaway food further compounding poor nutritional outcomes (Shu et al.). Association rule mining revealed that the convergence of takeaway dependence, inadequate sleep, and physical inactivity predicted unhealthy dietary patterns with over 90% confidence (Shu et al.). These findings directly inform workplace design by highlighting the need for on-site, unit-based meal distribution systems that align with shift workflows, thereby minimizing disruptive meal delays. Furthermore, environmental nudges such as traffic-light nutrient labeling in workplace cafeterias and the strategic restriction of high-sodium and high-sugar options can facilitate autonomous, healthier decision-making (Shu et al.). Complementary nutrition education tailored to irregular schedules, alongside accessible professional dietary counseling embedded within occupational health services, bridges the cognition-practice gap frequently observed in high-stress professions (Shu et al.).

Shift work, particularly prolonged night assignments, represents a critical occupational determinant of metabolic deregulations and cardiovascular risk. A comprehensive dose-response meta-analysis established that each 5-year increment in night shift duration corresponds to a 7% increase in cardiovascular disease incidence and a 4% rise in mortality risk, with fixed night schedules conferring substantially higher risk than rotating patterns (Xi et al.). This evidence mandates a structural redesign of shift scheduling protocols to prioritize circadian alignment. Workplace policies should minimize fixed night assignments, implement scientifically optimized rotation cycles, and mandate adequate recovery intervals between shifts to preserve sleep architecture (Xi et al.). Environmental design must further support circadian health through controlled lighting exposure, strategically timed meal breaks, and stress-reduction spaces that mitigate the physiological cascade of circadian disruption (Xi et al.). Such scheduling and environmental modifications directly address the biological pathways linking shift work to insulin resistance, systemic inflammation, and hypertension.

Routine occupational health examinations represent an underutilized platform for early metabolic risk detection and targeted intervention. Research documented a robust dose-response relationship between body mass index and multiple cardio metabolic risk factors among oilfield workers, with obesity associated with a seven-fold increased odds of hypertension and a three-fold increase in diabetes risk (Yu et al.). These findings advocate for the institutionalization of population-specific anthropometric thresholds and mandatory annual metabolic screenings within occupational health management systems (Yu et al.). Complementarily, sex-specific machine learning models incorporating routine biomarkers—such as BMI, age, and alanine aminotransferase, alongside sex-specific indicators like total cholesterol and uric acid for men or hemoglobin and hematocrit for women—achieved high predictive accuracy for metabolic syndrome (Wang et al.). Integrating such algorithmic risk stratification into workplace health surveillance enables proactive, precision-targeted interventions (Wang et al.). Routine monitoring of liver function and lipid profiles, coupled with sex-tailored nutritional and lifestyle counseling, transforms occupational health checkups from passive assessments into dynamic prevention platforms (Wang et al.).

Metabolic health cannot be divorced from musculoskeletal integrity, particularly in occupations characterized by prolonged sedentary exposure or reduced gravitational loading. Research revealed that extended diving experience was associated with significant reductions in leg bone mineral content and fat-free mass, with handgrip strength serving as a practical, non-invasive proxy for bone health deterioration (Véliz et al.). These findings, coupled with high baseline blood pressure and prevalent smoking and alcohol use among divers, underscore the necessity of integrating musculoskeletal monitoring with cardio metabolic screening in occupational settings (Véliz et al.). Workplace design should incorporate accessible resistance training facilities, promote weight-bearing physical activity breaks, and normalize the use of simple functional assessments like handgrip dynamometry as routine screening tools (Véliz et al.). Moreover, the synergistic relationship between physical activity, sleep quality, and dietary adherence supports the implementation of multi-component wellness infrastructure that simultaneously addresses movement, recovery, and nutrition (Shu et al.). Active workstations, structured exercise programs, and family-engaged health initiatives can disrupt the cycle of time poverty and metabolic decline commonly observed in demanding occupations.

The translation of these evidence-based strategies into sustainable practice requires organizational commitment and policy alignment. Workplace design initiatives must be embedded within broader occupational safety and public health frameworks, leveraging anonymized health data for continuous risk modeling while ensuring ethical data governance (Wang et al.). Cultivating a culture of health championed by educated senior staff, promoting family-involved dietary monitoring, and aligning institutional policies with national wellness initiatives create reinforcing ecosystems that sustain behavioral change (Shu et al.). Importantly, interventions must be dynamically refined through iterative data mining and machine learning applications, ensuring that workplace health programs evolve in response to emerging risk patterns and workforce demographics (Wang et al.; Shu et al.). Subgroup analyses further emphasize the importance of tailoring interventions: stronger BMI-hypertension associations among current smokers, stronger BMI-diabetes associations in older workers, and stronger BMI-low HDL-C associations in males suggest that targeted, stratified approaches may yield greater preventive impact (Yu et al.).

In conclusion, the convergence of recent occupational health research provides a robust empirical foundation for redesigning work environments to actively mitigate obesity and metabolic syndrome. By restructuring nutritional access, aligning shift schedules with circadian biology, embedding predictive health surveillance, integrating musculoskeletal-metabolic monitoring, and fostering supportive organizational policies, workplaces can transition from passive settings of health risk to active platforms of metabolic prevention. These design-driven strategies, grounded in sex-specific, age-stratified, and occupation-tailored evidence, offer a scalable and sustainable pathway to enhance workforce health, reduce chronic disease burden, and improve overall occupational resilience.

